# Nine quick tips for software containerization

**DOI:** 10.1371/journal.pcbi.1014197

**Published:** 2026-04-24

**Authors:** David Moreau, Kristina Wiebels

**Affiliations:** School of Psychology and Centre for Brain Research, University of Auckland, Auckland, New Zealand; Montreal, CANADA

## Abstract

Software containerization has become a cornerstone of modern computational biology, enabling researchers to package code, dependencies, and execution environments in portable and reusable units. Containers support reproducibility, facilitate collaboration, and lower barriers to deploying complex computational workflows across heterogeneous systems. At the same time, inappropriate or superficial use of containers can undermine these benefits, leading to brittle environments, security risks, or false confidence in reproducibility. In this article, we present nine practical and actionable tips for using software containers effectively in computational biology research. Rather than focusing narrowly on container syntax or tooling, we address conceptual decisions that arise throughout the research lifecycle: when containerization is appropriate, how to balance reproducibility with flexibility, how to manage dependencies and data, and how to share containers responsibly. These tips are intended for researchers with varying levels of experience, from those adopting containers for the first time to those maintaining mature, containerized workflows.

## Introduction

Computational biology increasingly depends on complex software stacks that combine programming languages, libraries, command-line tools, and system-level dependencies. Reproducing such environments across machines and over time has long been recognized as a major challenge for scientific reproducibility [1–5]. Software containers, most commonly implemented using technologies such as Docker, Apptainer (formerly Singularity), and other runtimes implementing the Open Container Initiative (OCI) specification, have emerged as a practical solution to this problem.

Containers encapsulate applications and their dependencies into lightweight, portable images that can be executed consistently across different systems. Unlike virtual machines, containers share the host operating system kernel, making them relatively efficient while still providing strong isolation of user-space environments [3,6]. In computational biology, containers are now routinely used to distribute bioinformatics tools, share complete analysis pipelines, support workflow systems, and deploy services in the cloud or on high-performance computing (HPC) infrastructure [7–10].

Despite their growing popularity, containers are not a panacea. Poorly designed container images can be opaque, insecure, difficult to maintain, or misleadingly non-reproducible. Moreover, containerization introduces new conceptual and practical decisions for researchers: what exactly should be containerized, how tightly versions should be pinned, how containers interact with data and workflows, and how long-term usability can be ensured.

**Fig 1:**
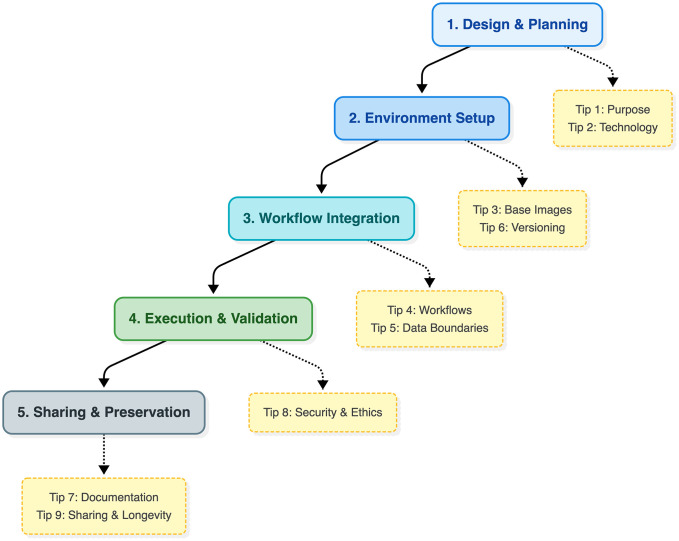
Containerization as a sequence of decisions across the computational research lifecycle. The diagram illustrates five stages of computational research practice, from initial design and environment construction to workflow integration, execution, and long-term sharing. The nine tips presented in this article are mapped onto these stages as key decision points. This lifecycle perspective emphasizes that containerization is not a single technical step but a series of strategic choices that influence reproducibility, maintainability, security, and reuse throughout a research project.

Several prior publications have outlined best practices for reproducible computational research and container usage [2,4,11,12]. These contributions have largely focused on technical guidance for writing container specifications or on the role of containers within reproducible workflows. The present article complements this literature by framing containerization as a lifecycle decision process embedded throughout the computational research workflow. Fig 1 maps these tips onto the computational research lifecycle, emphasizing that containerization involves decisions spanning design, implementation, execution, and long-term preservation. Rather than focusing primarily on container syntax or build mechanics, the nine tips highlight conceptual trade-offs that arise from project design through long-term preservation. This framing aims to help researchers integrate containerization strategically into scientific practice rather than treating it as a purely technical implementation step.

## Tip 1: Be clear about *why* you are containerizing

Containerization should be a means to an end, not an end in itself. Before creating or adopting a container, it is essential to articulate the primary motivation for doing so. Common reasons include improving reproducibility, simplifying installation for users, ensuring consistent execution across platforms, or enabling deployment on shared infrastructure such as HPC clusters or cloud services.

Different motivations imply different design choices. For example, a container intended to support *exact* reproduction of a published analysis may need aggressively pinned software versions and frozen dependencies [2,11]. In contrast, a container designed for *reuse* and extension by others may benefit from clearer documentation, modularity, and a degree of flexibility in dependency versions. Containers built for interactive exploration may prioritize usability and tooling, whereas those for automated workflows may emphasize minimalism and stability.

Explicitly stating the purpose of a container (ideally in accompanying documentation) helps guide decisions throughout its development and signals expectations to users. Without such clarity, it is easy to conflate goals (e.g., reproducibility versus upgradability) that are, in practice, in tension.

## Tip 2: Choose the right container technology for your context

Although Docker has become the most visible container platform, it is not always the most appropriate choice for computational biology workflows. Many HPC systems restrict or prohibit Docker due to its security model, which historically requires a daemon running with root privileges. In multi-user environments such as shared clusters, this raises concerns that a misconfigured container or daemon could allow users to gain unintended access to host resources. For this reason, HPC environments often favor alternatives such as Apptainer, which are designed to run containers without requiring elevated privileges [13,14].

When selecting a container technology, consider where and how the container will be executed. For local development and cloud deployment, Docker or Podman may be suitable. For shared clusters and national computing facilities, Apptainer is often the standard. Importantly, most modern container ecosystems are converging around the OCI image format, allowing images built with Docker-compatible tools to be executed by other runtimes with minimal modification [15].

Portability across runtimes is not automatic, however. Subtle differences in filesystem layout, user permissions, and supported features can affect behavior. Testing containers in their target execution environments is thus essential. Selecting a technology that aligns with your anticipated deployment context can avoid unnecessary friction and rework later in the research lifecycle.

## Tip 3: Start from well-maintained base images

Container images are rarely built from scratch. Instead, they typically extend a base image that provides an operating system and, in many cases, language runtimes or domain-specific tooling. Choosing a well-maintained base image is one of the most consequential decisions in container design.

Official images provided by trusted organizations (e.g., the Docker Official Images, the Rocker project for R, or BioContainers for bioinformatics tools) benefit from community oversight, regular updates, and documented maintenance practices [9,16,17]. These images reduce the risk of hidden vulnerabilities and outdated dependencies. For example, a container designed to run an RNA-seq quantification tool such as Salmon might extend a minimal Ubuntu or BioContainers base image that already provides stable system libraries and package management.

Regardless of the source, base images should be referenced using explicit version tags rather than mutable labels such as *latest*. However, it is important to recognize that tags themselves are mutable and can be reassigned by maintainers. For strict reproducibility, containers can also be referenced by their image digest, an immutable cryptographic identifier that always resolves to the exact same container image. Version pinning makes builds more predictable and aids future debugging and interpretation. At the same time, researchers should recognize that even versioned images may receive backward-compatible updates, and truly long-term preservation may require archiving image digests or exporting images to research data repositories [18].

## Tip 4: Integrate containers into workflows rather than treating them as isolated tools

In computational biology, containers rarely exist in isolation. They are typically embedded within larger workflows that include data preprocessing, analysis, visualization, and reporting steps. Treating containers as first-class components of these workflows improves both usability and reproducibility.

**Fig 2:**
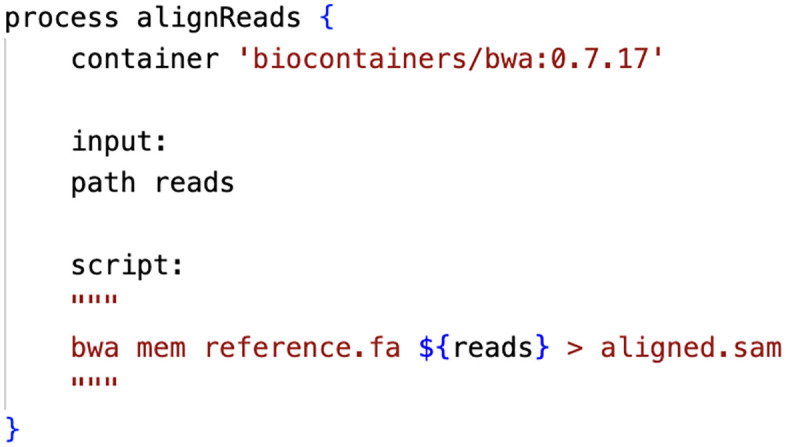
Example of container integration in a Nextflow workflow. This code snippet demonstrates how containers can be specified for individual processes within a workflow management system. The container directive isolates the alignment tool (here, BWA version 0.7.17) in its own environment; other pipeline steps can use different containers. This modular approach allows updating a single tool without rebuilding the entire computational environment, improving both maintainability and reproducibility.

Workflow management systems such as Snakemake, Nextflow, CWL, and WDL provide native support for executing individual steps within containers [8,10,19]. This approach allows different tools to be encapsulated separately, reducing image complexity and enabling clearer provenance tracking. For instance, a Nextflow pipeline might specify containers for individual processes (Fig 2). Here, the alignment step runs in an isolated environment with only the required alignment tool and its dependencies, whereas other pipeline steps can use different containers. This modularity means updating the alignment tool requires changing only one line, rather than rebuilding an entire monolithic image.

From this perspective, a container is best viewed as a reusable execution unit rather than a complete research environment. Designing containers to perform a well-defined task, with clear inputs and outputs, aligns with modular workflow principles and lowers barriers to reuse by others.

As a concrete example, consider a typical RNA-seq analysis pipeline consisting of quality control, read alignment, transcript quantification, and differential expression analysis. Each stage can be encapsulated in a separate container—for example, FastQC for quality control, STAR or HISAT2 for alignment, Salmon for transcript quantification, and DESeq2 for downstream statistical analysis. Workflow systems can orchestrate these containers sequentially, ensuring that each stage runs in a controlled environment while preserving modularity across the pipeline.

## Tip 5: Be explicit about data boundaries

A common source of confusion and error in containerized workflows concerns the handling of data. Containers should generally *not* embed large datasets or irreplaceable inputs directly within the image. Instead, data should be provided at runtime via mounted directories, object storage, or workflow-managed data channels [2,20].

**Fig 3:**
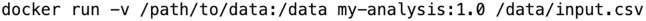
Mounting external data to a Docker container at runtime. This command illustrates the separation between software (inside the container) and data (external to the container). The -v flag binds the host directory/path/to/data to the container’s /data directory, making external files accessible to the containerized analysis without embedding them in the image. This approach keeps images small, portable, and free from data privacy or licensing concerns while maintaining clear provenance of required inputs.

Clear separation between software (inside the container) and data (external to the container) has several advantages. It keeps images small and distributable, avoids licensing or privacy issues associated with redistributing data, and makes it explicit which inputs are required to reproduce a result. In practice, this separation is implemented by mounting host directories at runtime. For example, when running a Docker container as shown in Fig 3, the -v flag makes the host directory/path/to/data available inside the container at /data, allowing the containerized software to access external data without embedding it in the image. Similarly, Apptainer automatically binds common directories like $HOME and $PWD, though explicit binding can be specified with --bind flags for clarity and portability across systems.

Mounting host directories is the most common strategy for providing data to containers, but several alternatives are increasingly used in bioinformatics workflows. Workflow engines such as Nextflow and Snakemake often stage input files automatically and manage data flow between containerized steps using explicit input/output channels. Cloud-based analyses may instead retrieve data dynamically from object storage systems such as Amazon S3 or Google Cloud Storage. In large-scale genomic workflows, reference datasets such as genome indexes are sometimes distributed via shared read-only volumes or cached datasets maintained by the workflow system. These approaches maintain the principle that containers encapsulate software rather than data while allowing flexible data provisioning across diverse computational infrastructures.

Remember, documenting expected data formats, directory structures, and environment variables is as important as packaging the software itself. Without this information, containers can become opaque “black boxes” that are technically runnable but scientifically unusable.

## Tip 6: Balance reproducibility with maintainability

One of the central tensions in containerization is between strict reproducibility and long-term maintainability. Freezing every dependency can help ensure that an analysis runs identically years later, but it can also lock in bugs, security vulnerabilities, and obsolete software [21].

Researchers should make this trade-off explicit. For published analyses, archiving an immutable container image that corresponds exactly to the reported results is often appropriate [11]. For ongoing projects or community tools, maintaining a living container that receives updates may better serve users.

Versioning strategies can help reconcile these goals. For example, semantic versioning allows container images to evolve while preserving stable releases. Accompanying documentation should clearly state the intended lifespan and update policy of a container.

In addition to semantic version tags, containers can also be referenced using image digests, which are immutable cryptographic hashes uniquely identifying a specific container image. Unlike tags, which may be reassigned by maintainers, digests always resolve to the exact same image. When strict reproducibility is required—such as reproducing the computational environment associated with a published analysis—referencing containers by digest provides stronger guarantees than relying on tags alone.

Some projects adopt a dual-tagging strategy to balance stability and ongoing development. For example, a container associated with a published analysis may be labeled with a fixed “publication” tag or release version, together with a rolling “latest” tag tracking ongoing development. Modern CI/CD platforms can automate this process by building and tagging container images whenever code changes are pushed to a repository, ensuring that version histories remain transparent while reducing the burden of manual version management.

## Tip 7: Document containers for humans, not just machines

A container that builds and runs successfully is not necessarily understandable. Documentation is essential for enabling others, including your future self, to interpret, trust, and reuse containerized environments. In bioinformatics workflows, such documentation might specify expected FASTQ formats, compatible genome assemblies, required reference datasets, or other domain-specific inputs necessary to run the containerized analysis correctly.

At a minimum, containers should be accompanied by human-readable documentation describing their purpose, contents, usage, and limitations. This may include a README file, inline comments in build recipes, and references to relevant publications or workflows. For complex images, higher-level summaries such as software bills of materials or environment manifests can be helpful [12].

Documentation should also state assumptions and known constraints, such as required hardware, expected runtime, or incompatibilities with certain systems. Treating documentation as an integral part of the container, rather than an afterthought, is crucial for sustainable scientific software.

## Tip 8: Consider security and ethics early

Containers are often perceived as inherently secure, but they can introduce risks if misused. Images may contain outdated libraries with known vulnerabilities, hard-coded credentials, or unnecessary privileges [22]. Several practical steps can mitigate these risks. First, regularly rebuild images from updated base layers, particularly when security patches are released. Tools like docker scan, Trivy, or Snyk can automatically detect known vulnerabilities in container images and their dependencies. For example, running *trivy image my-analysis:1.0* generates a report of Common Vulnerabilities and Exposures (CVEs) present in the image, allowing researchers to address high-severity issues before distribution.

Researchers should also be aware of potential software supply-chain risks, such as malicious or compromised base images obtained from public registries. Verifying image provenance and relying on trusted repositories can mitigate these risks. It is also important to recognize that vulnerability scanners depend on publicly reported CVEs and therefore may not detect newly discovered or unreported vulnerabilities. Automated scanning should therefore be treated as one layer of defense rather than a complete security solution.

Second, follow the principle of least privilege. Containers should not run as the root user unless absolutely necessary. Many container technologies now support rootless execution, where containers run with the permissions of the invoking user, substantially reducing security risks on shared systems [13,14]. When building images, explicitly set a non-privileged user (e.g., *USER researcher* in a Dockerfile) to avoid this pitfall.

Third, minimize the software installed in containers. Each additional package increases the attack surface and the likelihood of including vulnerable code. Multi-stage builds can help separate build-time dependencies from runtime requirements, producing smaller and more secure final images.

Ethical considerations are equally important, particularly when containers encapsulate workflows that process sensitive biomedical data. Although containers can help standardize secure execution environments, they do not eliminate the need for appropriate data governance, access controls, and compliance with legal frameworks such as GDPR or HIPAA [23]. Researchers should carefully evaluate whether containerizing a workflow might inadvertently facilitate unauthorized data access or circumvent institutional review board requirements.

For larger research teams or institutional platforms, continuous integration and deployment (CI/CD) pipelines can further strengthen security practices. Automated pipelines can rebuild container images when base images are updated, perform vulnerability scans, and enforce organizational security policies before containers are shared or deployed.

Finally, when sharing containers publicly, include explicit guidance about secure usage. Document any known security limitations, specify whether the container is intended for trusted or untrusted data, and warn against practices like embedding private credentials or running unvetted images with elevated privileges. Security and ethics are not afterthoughts; they should inform design decisions from the outset.

## Tip 9: Plan for sharing and longevity

Lastly, containerization delivers its greatest benefits when containers are shared and preserved in ways that support long-term access. Publishing container images alongside manuscripts, workflows, or software releases enhances transparency and reuse [2,24].

Several options exist for sharing containers, including public registries (e.g., Docker Hub, Quay.io), workflow repositories, and research data archives that issue persistent identifiers. Each option involves trade-offs in terms of discoverability, longevity, and citation. For containers that underpin published results, depositing an immutable image or recipe in a repository that provides a DOI can strengthen the scholarly record [18,25].

Planning for longevity also means recognizing that container technologies will continue to evolve. Preserving build recipes, documenting dependencies, and linking containers to version-controlled source code increases the likelihood that environments can be reconstructed or adapted in the future, even if specific tools become obsolete.

## Conclusion

Software containers have transformed how computational biology research is developed, executed, and shared. When used thoughtfully, they can substantially improve reproducibility, portability, and collaboration; when used uncritically, they risk becoming opaque artifacts that obscure rather than clarify scientific practice. The nine tips presented here emphasize deliberate decision-making over technical minutiae. Clarifying motivations, choosing appropriate technologies, integrating containers into workflows, and documenting and sharing them responsibly, can help researchers harness containerization as a robust scientific instrument rather than a fragile convenience. As container ecosystems continue to evolve, these principles provide a foundation for adapting new tools while maintaining core scientific values: transparency, reproducibility, and reuse.
